# LncRNAs-associated to genomic instability: A barrier to cancer therapy effectiveness

**DOI:** 10.3389/fgene.2022.984329

**Published:** 2022-11-21

**Authors:** Marco A Andonegui-Elguera, Rodrigo E. Cáceres-Gutiérrez, Diego Oliva-Rico, José Díaz-Chávez, Luis A. Herrera

**Affiliations:** ^1^ Unidad de Investigación Biomédica en Cáncer, Instituto de Investigaciones Biomédicas-Universidad Nacional Autónoma de México, Instituto Nacional de Cancerología, México City, Mexico; ^2^ Instituto Nacional de Medicina Genómica, Mexico City, Mexico

**Keywords:** lncRNAs, genomic instablity, cancer therapy, NORAD, CONCR

## Abstract

Although a large part of the genome is transcribed, only 1.9% has a protein-coding potential; most of the transcripts are non-coding RNAs such as snRNAs, tRNAs, and rRNAs that participate in mRNA processing and translation. In addition, there are small RNAs with a regulatory role, such as siRNAs, miRNAs, and piRNAs. Finally, the long non-coding RNAs (lncRNAs) are transcripts of more than 200 bp that can positively and negatively regulate gene expression (both in cis and trans), serve as a scaffold for protein recruitment, and control nuclear architecture, among other functions. An essential process regulated by lncRNAs is genome stability. LncRNAs regulate genes associated with DNA repair and chromosome segregation; they are also directly involved in the maintenance of telomeres and have recently been associated with the activity of the centromeres. In cancer, many alterations in lncRNAs have been found to promote genomic instability, which is a hallmark of cancer and is associated with resistance to chemotherapy. In this review, we analyze the most recent findings of lncRNA alterations in cancer, their relevance in genomic instability, and their impact on the resistance of tumor cells to anticancer therapy.

## Introduction

Most of the transcripts in the mammalian genome are non-coding. Within this group of transcripts are the long non-coding RNAs (lncRNAs), RNAs of more than 200 nucleotides that lack protein-coding potential (SamudyataCastelo-Branco and Bonetti, 2018). lncRNAs have nuclear or cytoplasmic localization. They can have different cellular functions by regulating the expression of coding genes, controlling protein modification, or serving as scaffolds for proteins that regulate chromatin structure. Due to their versatility, lncRNAs have been associated with different cellular processes, such as proliferation, differentiation, embryogenesis, stemness, regulation of genome stability and pathological processes such as carcinogenesis (SamudyataCastelo-Branco and Bonetti, 2018; Taniue and Akimitsu, 2021).

Genomic instability is a critical feature in cancer cells. It has been described as an enabling hallmark of cancer because it allows cell plasticity to acquire different cancer features (Hanahan and Weinberg, 2011). In addition, genomic instability is associated with increased aggressiveness and resistance to cancer therapy. It has been proposed that genomic instability confers heterogeneity to tumors so different clones can evolve, promoting drug resistance and tumor progression (Vargas-Rondon et al., 2017; Sansregret et al., 2018; Turajlic et al., 2019). The origin of genomic instability in cancer is not well defined. Chromosomal instability (defined as a high rate of changes in chromosome number and structure) has been associated with alterations in kinetochore-microtubule binding, centrosome duplication, and alterations in the expression of specific mitotic genes, tetraploidization events, defects in chromatid cohesion and telomere dysfunction (Tanaka and Hirota, 2016).

On the other hand, genes involved in detecting, repairing, and responding to DNA damage are mutated in different tumors. Germline mutations in these genes are associated with genomic instability syndromes that significantly increase the risk of developing cancer (Hanahan and Weinberg, 2011). As the lncRNAs are involved in a myriad of cell activities, it is not surprising that lncRNAs are associated with genomic instability in cancer. Although not all genomic stability-related lncRNAs have been associated with resistance in cancer therapy, their dysfunction and the consequent boost of genomic instability may result in resistance and progression in different tumors. In this review, we will discuss the different lncRNAs associated with genome maintenance, their alterations in cancer, and the possible repercussions on response to therapy and prognosis in cancer.

### LncRNAs at the chromosome stability

#### NORAD

**FIGURE 1:**
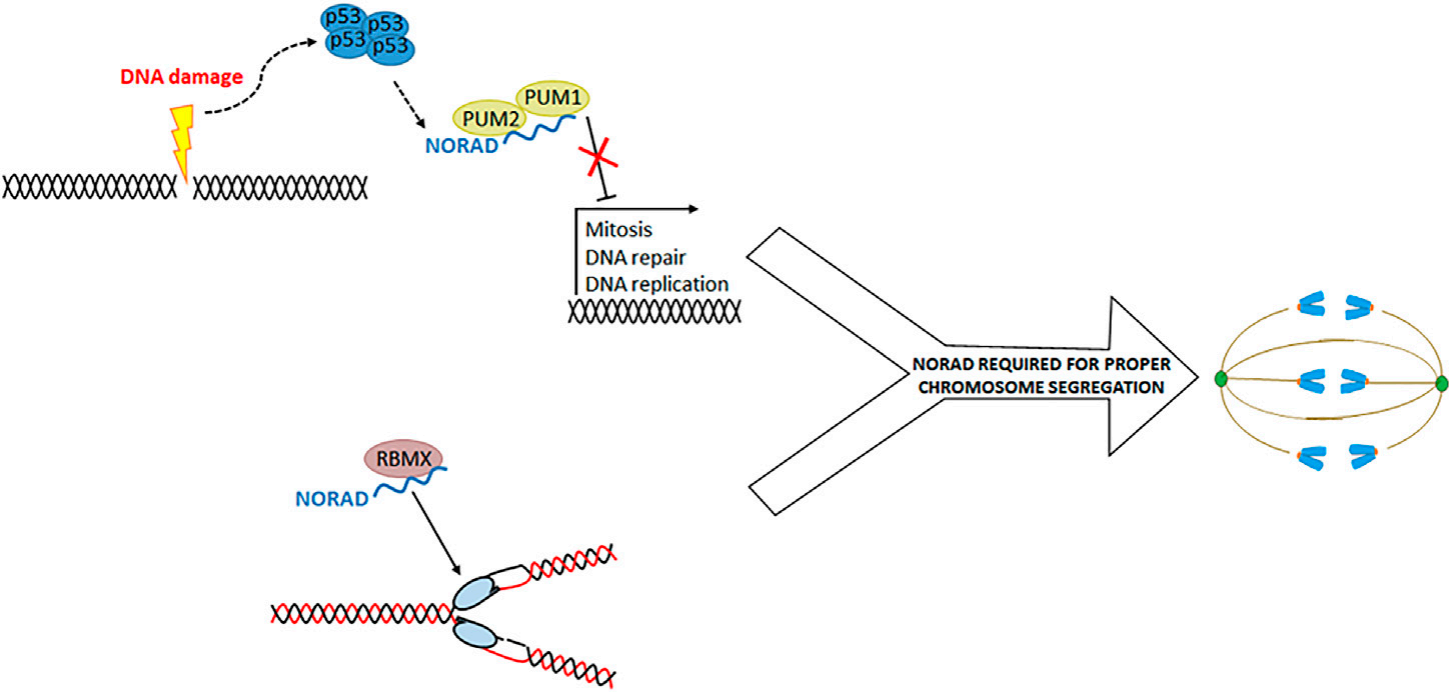
NORAD is involved in the proper segregation of chromosomes. Two mechanisms by which NORAD promotes chromosome segregation have been proposed. First (above), NORAD negatively regulates PUM1 and PUM2 proteins allowing the expression of genes involved in cell cycle processes. On the other hand, NORAD forms a ribonucleoprotein complex with the RBMX protein, where they have a role in DNA duplication and repair. However, it is not clear how this function participates in chromosome segregation.

Non-coding RNA activated by DNA damage (NORAD) is a lncRNA of approximately 5.3 kb expressed in different tissues and highly conserved in mammals (Tan et al., 2019). NORAD expression increased upon DNA damage in a p53-dependent manner, despite having no apparent p53 response elements (Lee et al., 2016; Soghli et al., 2021). Deletion of NORAD causes tetraploidization and mitotic defects, such as anaphase bridges and mitotic slippage (Lee et al., 2016)**.** Two mechanisms by which NORAD maintains genomic stability have been proposed. First is the binding of NORAD to PUMILIO RNA binding proteins (PUM1 and PUM2). PUM proteins bind RNA and inhibit the expression of several genes, including genes related to mitosis, DNA repair, and replication (Elguindy et al., 2019). NORAD binding to PUM prevents repression of these genes and maintains genomic stability (Figure 1).

On the other hand, NORAD purification and quantitative liquid chromatography-mass spectrometry demonstrated that NORAD binds to the RBMX protein (whose knockdown provokes DNA repair and sister-chromatid separation defects) (Munschauer et al., 2018)**.** NORAD and RBMX are part of a ribonucleoprotein complex involved in DNA replication and repair, and NORAD depletion reduced replication fork velocity (Figure 1). There is debate about which of these mechanisms is more relevant to genome maintenance. In a cell model where NORAD has been deleted, it has been shown that expression of wildtype NORAD or a NORAD fragment without the RBMX binding site can reverse the generation of aneuploidy or the formation of alterations during segregation (Elguindy et al., 2019). They conclude that RBMX is a dispensable protein for NORAD genome maintenance activity. However, the functionality of NORAD-RBMX may be related to DNA replication and not mitosis. Therefore, both mechanisms are not mutually exclusive and can be an essential part of NORAD activity.

Because of NORAD activity in genome maintenance, its downregulation or deletion can be relevant to the development and prognosis of malignant tumors. Many published studies have been concerning its role in cancer development and prognosis. However, most investigations have associated increased NORAD levels with the acquisition of malignant features or a worse disease prognosis. NORAD overexpression has been found in breast, stomach, liver, pancreas, breast, bladder, melanoma, colon, prostate, lung, endometrium, ovary, and cervix tumors, as well as glioma and neuroblastoma. The oncogenic activity of NORAD is mediated by pathways such as TGFb, MAPK, Akt/mTOR, etc., and the function of NORAD as a microRNA inhibitor by sponging microRNAs. There are recent reviews for details about these findings (Ghafouri-Fard et al., 2021; Soghli et al., 2021)**.**

However, few studies have found a decrease in NORAD and a relationship with clinical parameters in malignant tumors. Yu et al., reported a decreased NORAD expression in endometrial cancer tumor tissue compared with normal tissue from TCGA data. In addition, they describe an association between low NORAD levels and shorter overall survival. In endometrial cancer samples (*n* = 56), they found an association between decreased NORAD and increased clinical stage. Because NORAD downregulation in endometrial cancer-derived cell lines promoted apoptosis, they focused on the involvement of FUBP1 (a NORAD-binding protein) in NORAD-driven apoptosis. FUBP1 is a protein that negatively regulates the transcription of pro-apoptotic genes. Thus, NORAD binding to FUBP1 promotes apoptosis (Han et al., 2020). Surprisingly, the role of NORAD in genome stability was not assessed in this study, so it will be necessary to determine whether the NORAD relevance in endometrial cancer is related only to the regulation of apoptosis or also to the control of genome stability. From NCBI’s Gene Expression Omnibus (GEO) data, a lower expression of NORAD was found in tumors compared to normal tissues in lung and breast tumors. Besides, there is a correlation between decreased NORAD expression and poor survival. In cohorts of lung adenocarcinoma samples (*n* = 95) and breast cancer samples (*n* = 70), NORAD was decreased in tumor samples vs. adjacent tissue. In both cohorts, a correlation was demonstrated between decreased NORAD and the presence of metastatic lymph nodes. In the same study, NORAD inhibited invasion and metastasis by sequestering the S100P (invasion and metastasis promoter) protein (Tan et al., 2019)**.** Again, genomic instability was not determined. Mice injected with MDA-MB-231 cells (which showed chromosomal instability) showed decreased metastatic capacity when chromosomal instability was reduced by overexpression of KIF2B or MCAK. Demonstrating that chromosomal instability promotes the invasive and metastatic phenotype (Bakhoum et al., 2018). Therefore, in addition to the role of S100P, the downregulation of NORAD may promote metastasis formation through chromosomal instability. Finally, another study found an association between low NORAD levels with higher stage and worse survival in neuroblastoma patient databases. The association between low NORAD levels and tumor stage was also found in a group of neuroblastoma patient samples (*n* = 40). *In vitro* studies with neuroblastoma-derived cell lines determined that decreased NORAD promotes cell proliferation, migration, and expression of DNA damage markers. Interestingly, NORAD downregulation was associated with reduced expression of chromosome segregation genes, such as SMC1A, RAD21, ESPL1, and PLK1 (Yu et al., 2020). This is the only study linking NORAD downregulation with clinical parameters and chromosomal instability in cancer tumors.

The role of NORAD in cancer biology is complex. Most studies demonstrate increased NORAD expression in tumors related to different pathways and microRNA regulation. However, it will be necessary to determine whether elevated levels of NORAD can modify genome stability. In this regard, it has been reported that A549 cells exposed to PM10 particles overexpress NORAD and Spindle Assembly Checkpoint (SAC) genes (MAD2L1, MAD1L1, BUB1B), and NORAD inhibition counteracts the overexpression of SAC genes (Santibanez-Andrade et al., 2021). Moreover, NORAD downregulation may be relevant for some specific tumors, such as lung, breast, endometrial, and neuroblastoma. Therefore, it is necessary to determine whether genomic instability caused by NORAD downregulation plays an essential role in the biology of these neoplasms. In addition, it is critical to assess whether NORAD downregulation is related to resistance to therapies used in these tumors in both clinical and *in vitro* studies.

#### CONCR

The lncRNA CONCR (cohesion regulator non-coding RNA), also known as DDX11-AS1, binds to the helicase DDX11 and participates in DNA replication by maintaining cohesion between sister chromatids. Depletion of CONCR results in the loss of sister chromatid cohesion. CONCR expression is indirectly repressed by p53. Overexpression of CONCR has been observed in different types of cancer, such as hepatocellular carcinoma, colorectal cancer, osteosarcoma, bladder cancer, gastric cancer, glioma, and non-small cell lung cancer. Its expression has been associated with tumor stage, recurrence, and lymph node metastasis (Tian et al., 2019; Feng et al., 2020a; Zhang et al., 2020a; Feng et al., 2020b; Xiang et al., 2022). CONCR activity is relevant for cell proliferation, as its decrease is associated with the inhibition of proliferation and increased cell death (Marchese et al., 2016). The oncogenic activity of CONCR is complex and has been associated with the negative regulation of different miRNAs and the activation of oncogenic pathways such as PI3K/AKT and Wnt/beta-catenin (Feng et al., 2020a; Xiang et al., 2022).

Due to the role of CONCR in tumor biology, it has been proposed as a therapeutic target (Shi et al., 2017). *In vitro* and *in vivo* models have shown that CONCR knockdown sensitizes paclitaxel-resistant breast cancer cells and oxaliplatin-resistant gastric cancer cells, respectively (Zhang et al., 2019; Song et al., 2020). Although breast cancer cells sensitization was associated with increased miR497 expression, it is possible that the increase in segregation errors due to CONCR knockdown, coupled with paclitaxel activity on microtubules, may allow mitotic defects to be increased and thus favor cancer cell death.

#### CCAT2

On the other hand, the overexpression of a lncRNA called CCAT2, which is conserved in mammals, was described in colorectal cancer samples (Redis et al., 2013). CCAT2 expression was elevated in colorectal cancer samples compared to adjacent tissue. In addition, tumors with microsatellite stability showed a higher CCAT2 expression than those with microsatellite instability. Overexpression of CCAT2 in cell lines and xenografted tumors promoted increased proliferation and metastasis. The oncogenic characteristics of CCAT2 are associated with MYC regulation (Redis et al., 2013; Pirlog et al., 2021). Moreover, in an analysis of HCT116 cell clones overexpressing CCAT2, multiple structural and numerical chromosomal alterations (aneuploidy and polyploidy) were found. It has also been shown that CCAT2 stabilizes BOP1 (a ribosomal protein), which increases the activity of AURORA B, a phenomenon associated with chromosomal instability (Chen et al., 2020).

In different *in vitro* models, it has been shown that the lncRNA CCAT2 promotes resistance to treatment with different drugs, such as 5-fluorouracil, platinum drugs, tamoxifen, and doxorubicin, among others. However, it is unclear whether this resistance can also be explained in patients. Furthermore, it is not well defined whether the resistance is due to its role in instability or is independent of it.

### lncRNAs from the centromere

**FIGURE 2:**
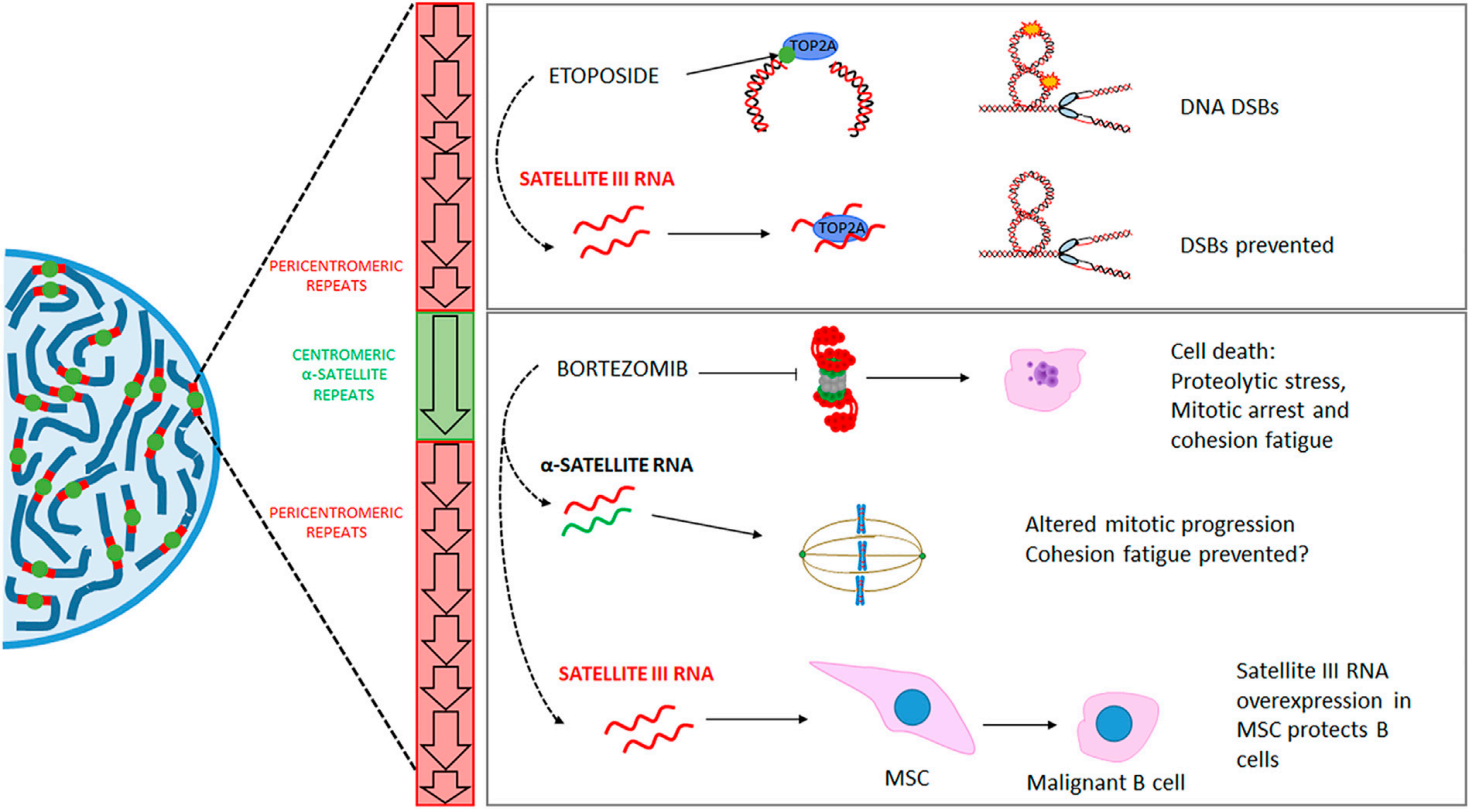
Mechanisms proposed to mediate the relationship between RNAs transcribed from the centromere and pericentromere and treatment response. Upper right panel: etoposide promotes DNA double-strand breaks (DSBs) through its interaction with topoisomerase 2 A (TOP2A) and the formation of a ternary complex with the DNA. Etoposide-induced satellite III RNAs participate in sequestering TOP2A in nuclear stress bodies, which prevents the generation of DNA DSBs. Lower right panel: Proteasome inhibition by bortezomib (used in treating multiple myeloma, among other malignancies) promotes cell death through proteolytic stress and a prolonged mitotic, followed by cohesion fatigue (among other mechanisms). Upon bortezomib treatment, delayed mitotic progression is associated with the overexpression of α-satellite RNAs. These transcripts interact with cohesin subunits, which could inhibit the establishment of cohesion fatigue. On the other hand, the expression of satellite III RNAs in mesenchymal stromal cells (associated with B-lymphocytes in the bone marrow) triggered by bortezomib has been shown to protect malignant B cells (the target of bortezomib) from bortezomib toxicity. DSB: Double Strand Breaks. MSC: Mesenchymal Stromal Cell.

The centromere is the genomic region upon which the kinetochore, the interface between the chromosomes and microtubules essential for chromosome segregation, is assembled (Fukagawa and Earnshaw, 2014). The centromere is epigenetically defined by the presence of the H3 variant histone CENP-A and accompanying histone post-translational modifications (Fukagawa and Earnshaw, 2014). At the genetic level, this region is populated by non-coding elements termed α-satellites, which are repeated in a head-to-tail orientation for up to several megabases in arrays known as Higher Order Repeats (HORs). However, not all α-satellite repeats make part of a HOR or host the CENP-A variant histone. Instead, the kinetochore is usually assembled on the largest HOR of each chromosome, and the flanking α-satellite repeats are part of a structure named pericentromere (Altemose et al., 2022). The pericentromere is a large region that contains several other repetitive elements and has its own epigenetic (primarily repressive) features. In the literature, the term “centromere” has been used somewhat interchangeably to refer to the core centromere or to entail the centromeric and pericentromeric regions. Historically, the pericentromere and the centromere core have been difficult to discern. Furthermore, both structures have been considered transcriptionally inert. However, this is not the case. Although the centromere and the pericentromere bear epigenetic repressive marks, they can also display histone post-translational modifications associated with active chromatin. Basal centromeric expression is detectable in normal human cells from different tissues (Eymery et al., 2009), even by Northern blot (Caceres-Gutierrez et al., 2022). But these regions are also transcriptionally dynamic and highly responsive to the cellular context and internal and external stimuli, such as cell cycle progression (Bury et al., 2020), differentiation (Bouzinba-Segard et al., 2006), cancer progression (Ting et al., 2011; Zhu et al., 2011), heat shock, osmotic pressure, oxidative stress, and exposure to heavy metals (Valgardsdottir et al., 2008). The lncRNAs transcribed from the centromere or pericentromere have roles in the cell. In the specific context of cancer treatment, the expression of centromeric and pericentromeric regions in response to chemotherapeutic agents can alter cellular behavior, impacting treatment response. This has been demonstrated for the DNA damaging agent etoposide. Early evidence showed that several genotoxic agents cause overexpression of centromeric repeats in non-cancerous murine cells (Hedouin et al., 2017). Further study in humans demonstrated satellite III repeat hypomethylation in cancerous compared to normal tissue, which was associated with etoposide resistance in non-small cell lung carcinoma (Kanne et al., 2021). The authors also showed that etoposide resistance is accomplished by sequestering topoisomerase 2A (TOP2A) in nuclear stress bodies. The seizing of TOP2A by nuclear stress bodies prevents TOP2A from forming a complex with etoposide, which would promote DNA damage. Therefore, pericentromeric transcription stimulates tumor resistance to etoposide in this model (Figure 2).

In this regard, we have demonstrated that proteasome inhibitors promote the overexpression of several repetitive RNAs, including the centromeric α-satellites (Caceres-Gutierrez et al., 2022). Centromeric and pericentromeric lncRNAs have also been associated with resistance to different antineoplastic drugs, such as the proteasome inhibitor bortezomib (Figure 2). Our study demonstrated that the upregulation of α-satellite DNA alters mitotic progression. Moreover, work from another group showed that the bortezomib-induced upregulation of satellite III (Figure 2) DNA in mesenchymal stromal cells protected multiple myeloma cells from bortezomib-induced cells (Enukashvily et al., 2022).

Therefore, centromeric and pericentromeric lncRNAs alter cellular behavior with negative consequences for cancer treatment. However, further research will be necessary to determine whether the exact mechanisms observed *in vitro* operate *in vivo* and *vice versa* to reconstruct a complete panorama of centromeric and pericentromeric transcription and its impact on treatment outcomes. Such a research effort would help provide clues to improve the outcome for cancer patients.

### TERRA-telomere homeostasis and genomic stability

Given their linear nature, the homeostasis of human chromosomes calls for the uninterrupted surveillance of chromosome termini. For this reason, the telomere assembles at the ends of linear chromosomes. Telomeres are specialized nucleoprotein complexes that maintain the integrity of the chromosome, promoting the homeostasis of the whole molecule in interphase and ensuring appropriate chromosome segregation during mitosis (Chuang et al., 2004; Azzalin et al., 2007; Heidenreich and Kumar, 2017). Capping telomere ends, loop formation, strand invasion, chromatin compaction, and establishment of guanine quadruplexes are necessary to maintain telomere integrity. The Telomeric repeat-containing RNA (TERRA) is a lncRNA that takes part in the previously listed processes.

To prevent the DNA repair machinery from recognizing and processing the ends of linear chromosomes, the protein complex Shelterin assembles on the telomeric repetitive track and aids in the formation of telomere loops (T-loops) (Blasco, 2005). Together, Shelterin deposition and T-loop formation constitute telomere capping. At the end of DNA replication, newly synthesized telomeres are uncapped and must be protected from nucleolytic degradation. TERRA aids in the re-establishment of the Shelterin complex by directly associating with TRF2 (Mei et al., 2021) and by associating with the Heterogeneous nuclear ribonucleoprotein A1 (hnRNPA1) and then mediating an exchange between RPA and Protection of telomeres protein 1 (POT1) (Flynn et al., 2011). By ensuring the formation of the Shelterin complex, TERRA prevents the abnormal shortening that would take place through 5′–3′ nucleolytic degradation (Longhese et al., 2010).

Telomeres shorten at a regular rate as the cells divide and age. As these sequences become shorter, constitutive heterochromatin is lost at telomeres and sub-telomeres; this promotes TERRA transcription in chromosome arms with short telomeres (Yehezkel et al., 2008). In the presence of human telomerase reverse transcriptase (hTERT), increased TERRA transcription ensures hTERT recruitment onto critically short telomeres (Farnung et al., 2012). This prompts telomere extension, heterochromatin recovery, and the eventual reduction of TERRA expression at the extended telomeric locus (Wang et al., 2015; Oliva-Rico et al., 2022).

Despite the positive effects of TERRA transcription in telomere recovery, chromosome stability requires that TERRA expression return to its normal rate. Otherwise, the accumulation of this lncRNA can become detrimental (Aguilera and Garcia-Muse, 2012; Arora and Azzalin, 2015). DNA-RNA hybrids and the DNA strand they displace are called RNA loops (R-loops). To allow the progression of the replication fork Telomere R-loops (TRLs) must be resolved during S-phase. Otherwise, the stalled polymerase leads to fork collapse and double-strand breaks (Balk et al., 2014). Furthermore, in the telomeric track, DSB can lead to the accelerated shortening of telomeres. Therefore, overexpression of TERRA would result in more TRLs at the transcribing loci, further shortening an already critically short telomere.

The significance of TERRA expression in cancer is not well understood. Reported that in the presence of telomerase, the hypomethylation-induced steady expression of TERRA allowed the extension of the telomeres associated with hypomethylated loci in a human colon cancer cell line (Nergadze et al., 2009; Farnung et al., 2012). However, Oh et al. reported that in tissue from patients with hepatocellular carcinoma, telomeres could be extended in chromosomes with both hypo and hypermethylated subtelomeric loci, making TERRA expression seem inconsequential for telomere elongation in liver cells (Oh et al., 2011). Arnoult et al. then found that in fibrosarcoma and lung cancer-derived cell lines and non-tumoral fibroblast telomere extension directly silenced TERRA expression at the associated subtelomeric loci by increasing the levels of H3K9me3 (Arnoult et al., 2012). However, in gastric cancer, breast cancer, and cervical carcinoma-derived cell lines, Smirnova et al. found that telomere length and TERRA expression did not correlate (Smirnova et al., 2013).

It is clear that the transcriptional regulation of TERRA expression is tissue-specific. There seem to be stark differences between the results obtained from directly analyzing tissue samples and those results from cell lines of the same tissue lineage. Moreover, the expression of TERRA at each telomere appears to be regulated in a telomere-specific way, making it hard to pinpoint a reliable regulation pathway for the eventual use of this lncRNA as a biomarker for the prognosis of the disease.

It has proven complicated to directly relate TERRA expression and the possible consequences of an altered telomere length, such as cell-life span, disabled tissue replenishment, degenerative disorders (Maicher et al., 2014), tumor aggressiveness (Deng et al., 2012), and radiation sensitivity (Smirnova et al., 2013), to mention a few. However, there is a clear association between TERRA expression and the proliferation rate of a cell. Flynn et al. have proposed that altering the transcriptional control of TERRA could induce chromosome fragmentation and apoptosis, thus serving as a therapeutic strategy (Flynn et al., 2015). But there is still an absence of a well-established approach to depleting TERRA levels (Gala and Khattar, 2021). Given that TERRA expression is cell cycle-regulated, such a treatment’s effectiveness would likely depend on functional checkpoints. We consider that an adequate TERRA-mediated treatment should not focus on the transcription of the lncRNA but rather on the effects of its accumulation. The primary goal of this TERRA-focus treatment would be to indirectly induce accelerated telomere attrition, hinder cell division and induce either cellular senescence or mitotic catastrophe; this scenario could slow tumor growth and temporarily reduce cancer aggressiveness.

**FIGURE 3:**
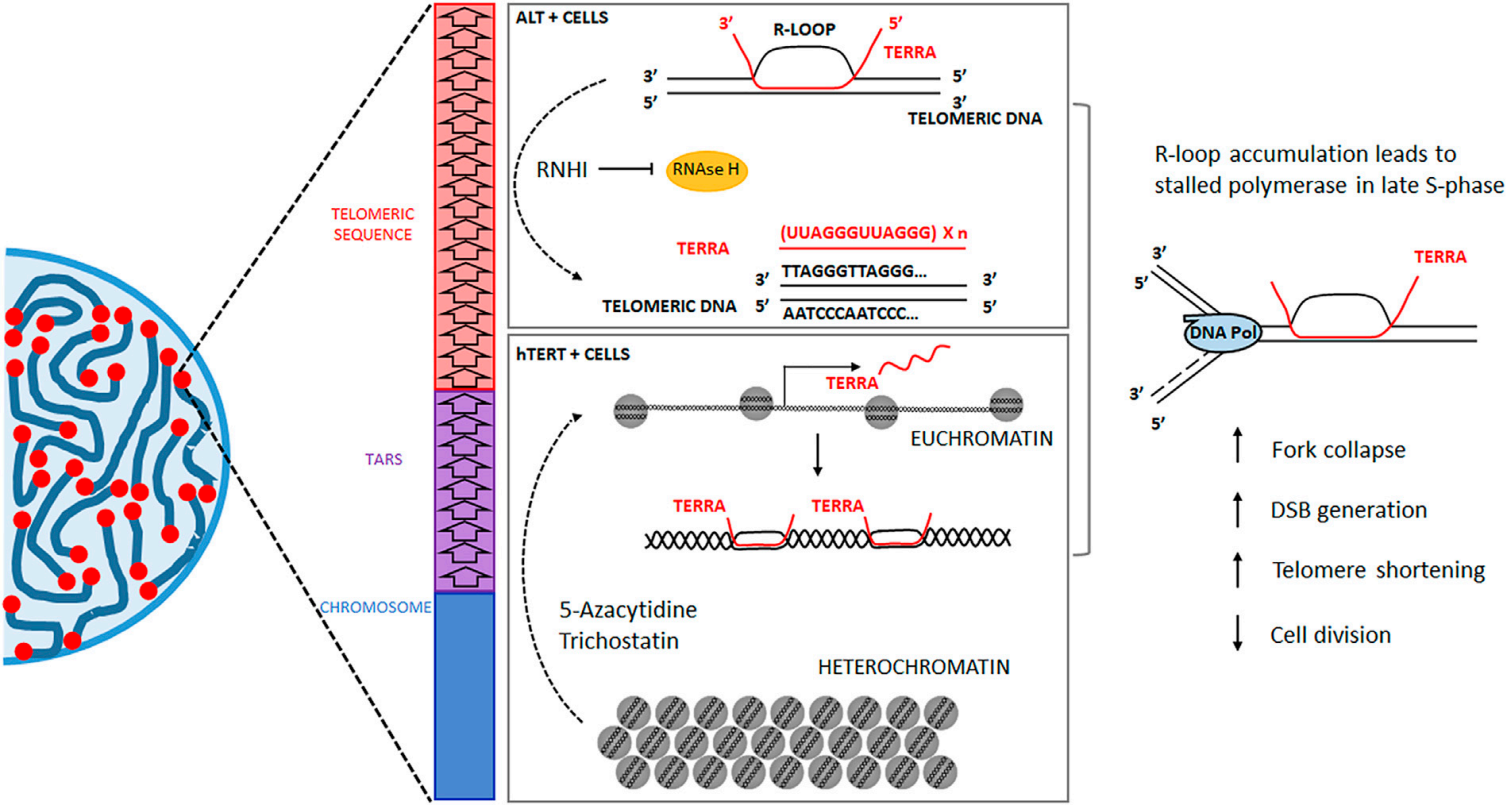
The telomeres in eukaryotic cells protect the extremes of the linear chromosomes. Note that the canonical telomeric sequence (5′-TTAGGG-3′) degenerates into associated repeats towards the chromosome sequence. The lncRNA TERRA forms R-Loops in the telomeric tract to increase telomere protection. Still, a potential therapeutic application can derive from R-Loop accumulation by inducing senescence/apoptosis. hTERT + tumor cells treated with 5-azacytidine or trichostatin will increase TERRA transcription; ALT + tumor cells treated with RNHIs will not be able to dismantle DNA/RNA hybrids. Both treatments can lead to telomeric R-Loop accumulation, DNA damage, telomere shortening, and arresting cell division.

It is essential to consider which telomere maintenance mechanism is active before using any telomere-focused therapy because the expression of hTERT or homologous recombination in ALT cells can influence the outcome of those treatments (Oliva-Rico and Herrera, 2017; Gala and Khattar, 2021). TERRA expression is already upregulated in ALT-dependent tumor cells, evidencing its oncogenic role (Azzalin et al., 2007; Gala and Khattar, 2021), so a practical approach like reducing the effect to the ribonuclease RNaseH1 or depleting its expression (Figure 3), would favor the buildup of TRLs (Arora et al., 2014). These structures already occur in human cells under physiological levels of TERRA expression (Toubiana and Selig, 2018). Therefore, under tumorigenic conditions, the elevated transcription of TERRA is more likely to trigger the deleterious effects of TRLs in the cells with a higher proliferation rate. In telomerase-dependent tumor cells, TERRA can behave as a tumor suppressor, and therefore, its expression is considerably lower (Azzalin et al., 2007; Gala and Khattar, 2021); in these cells, TERRA transcription can be prompted by the use of Trichostatin A or 5-aza cytidine (5-AZC) (Figure 3), both drugs reported to induce an accumulation of TERRA (Azzalin and Lingner, 2008).

### lncRNAs in regulating the response to DNA damage

#### PANDA

**FIGURE 4:**
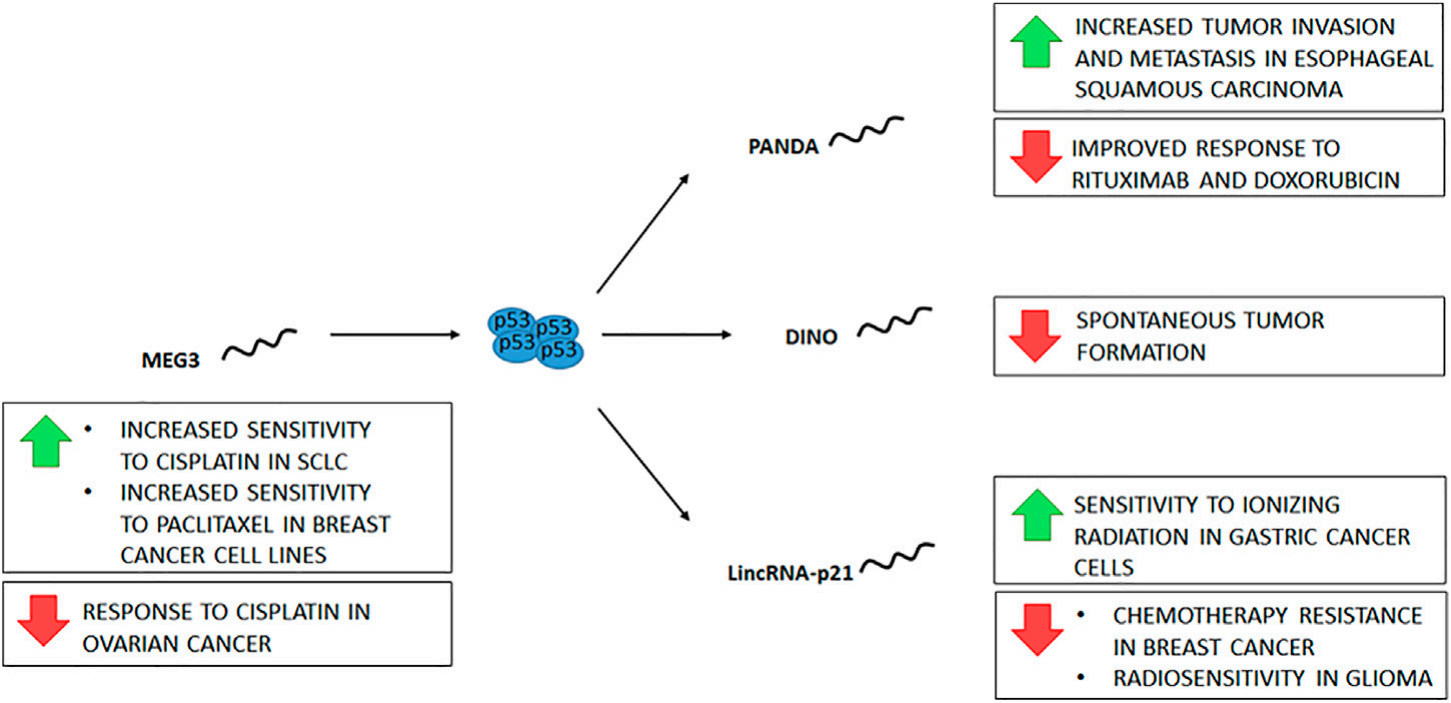
lncRNAs participate in the p53 pathway downstream and upstream of p53. Four lncRNAs are depicted relative to p53, along with the identified consequences of their expression (green arrows) or repression (red arrows), be it experimental or naturally occurring in cancer.

Genome maintenance requires DNA surveillance for the detection and repair of DNA damage. p53 is considered the guardian of the genome because it participates in different mechanisms of the DDR. Recently, different lncRNAs have been described whose function is directly related to the activity of p53. The p21-associated ncRNA DNA damage activated (PANDA) is a transcriptional target of p53 expressed in response to DNA damage (Hung et al., 2011; Shi et al., 2019). PANDA inhibits apoptosis by preventing the transcription factor NF-YA from binding to its targets, which include apoptotic genes (Hung et al., 2011). In addition, the interaction of PANDA with SAFA (an RNA- and DNA-binding protein) promotes cell proliferation through the activity of cyclins D1/2 and E1 (Shi et al., 2019). It has been proposed that PANDA regulates proliferation and senescence by forming complexes with proteins. In proliferating cells, it binds to the SAFA protein and negatively regulates different genes, including p21 and PANDA expression. On the other hand, when senescence is promoted, PANDA binds to the NF-YA factor inhibiting apoptosis of senescent cells (Puvvula et al., 2014). As mentioned above, the function of PANDA can be antagonistic according to the cellular context. Likewise, its expression is either decreased or increased in different tumor types. Some studies have found decreased PANDA levels relative to adjacent tissue in cellular hepatocarcinoma tumors (Puvvula et al., 2014; Peng et al., 2017), while others have demonstrated overexpression (Peng and Fan, 2015). Despite the conflicting data, both overexpression and underexpression of PANDA may contribute to tumor development and aggressiveness. PANDA down-regulation has been described in breast tumors, lung cancer, lymphoma, papillary thyroid carcinoma, and gastric cancer (Wang et al., 2017a; Esfandi et al., 2019; Zhang et al., 2020b; Ghafouri-Fard et al., 2022; Islam et al., 2022). On the other hand, an increase in PANDA expression has been reported in colorectal cancer tumors, glioma, thyroid gland carcinoma, renal cell carcinoma, cholangiocarcinoma, osteosarcoma, bladder cancer, and cervical cancer (Huang et al., 2017; Zou et al., 2018; Rivandi et al., 2019; Qing et al., 2021; Guo et al., 2022). In diffuse large B-cell lymphoma, an association was found between PANDA down-regulation and response to rituximab (Wang et al., 2017a; Ghafouri-Fard et al., 2022). Furthermore, in cell lines, PANDA knockdown was also found to sensitize cells to doxorubicin treatment (Hung et al., 2011). Moreover, in esophageal squamous carcinoma tissue, a higher expression of PANDA was reported compared to adjacent tissue related to tumor invasion, metastasis, and stage (Figure 4). This finding was associated with SAFA regulation by PANDA (Shi et al., 2019). Despite the evidence of PANDA regulation by p53, it is unclear whether it functions in the regulation of apoptosis and proliferation or may be involved in other processes of DNA damage responses.

#### DINO

The expression of the Damage Induced Noncoding (DINO) RNA is also regulated by p53; furthermore, DINO stabilizes p53 and participates in the DNA damage response by regulating the expression of p53 targets (Schmitt et al., 2016). *In vivo* models have shown that DINO deletion promotes the development of spontaneous tumors independently of p53 status (Figure 4). Furthermore, tumors caused by DINO knockout are tissue-specific (Marney et al., 2022). Using the TCGA Pan-Cancer database, it was found that many tumors have methylation in the CpG shore downstream of the DINO TSS associated with lower expression, which would explain the low mutation rate in this gene (Marney et al., 2021). In tumors of patients with gastric cancer, DINO is downregulated compared to adjacent tissue (Liu et al., 2019).

#### LincRNA-p21

The lncRNA lincRNA-p21 is another p53-regulated RNA. It was described as a lncRNA that participates in the p53 pathway by repressing specific genes in complex with hnRNP-K (Huarte et al., 2010). However, many functions have been described for lincRNA-p21, such as direct interaction with MDM2 (a negative p53 regulator) and p53, transcription of p21, cell proliferation regulation, metastasis, and intercellular communication (Dimitrova et al., 2014; Huang et al., 2022). LincRNA-p21 is inhibited in different malignant tumors, including colorectal carcinoma, breast cancer, cervical carcinoma, skin cancer, hepatocellular carcinoma, non-small cell lung carcinoma, head, and neck squamous cell carcinoma, gastric cancer, prostate cancer, and chronic lymphocytic leukemia (Wang et al., 2017b; Wang et al., 2017c; Huang et al., 2022). Estrogen receptor alpha (ER-alpha) mediated downregulation of lincRNA-p21 has been associated with chemotherapy resistance in breast cancer (He et al., 2021). *In vitro* studies have shown that lincRNA-p21 expression enhances the sensitivity of gastric cancer cells to ionizing radiation (Chen et al., 2019). However, another study showed that lincRNA-p21 knockdown promoted radiosensitivity in glioma cells (Figure 4) (Shen et al., 2017).

#### MEG3

Other lncRNAs can act upstream of p53 and regulate its function. Maternally expressed gene 3 (MEG3) is a lncRNA that regulates p53 directly and indirectly. MEG3 inhibits the binding of p53 to MDM, promoting p53 stability and, on the other hand, stimulates p53 expression (Ghafouri-Fard and Taheri, 2019). MEG3 is downregulated in different tumors, such as breast, liver, glioma, colorectal, cervical, gastric, lung, ovarian, osteosarcoma, kidney, bladder, prostate, melanoma, retinoblastoma, thyroid, leukemia, and lymphoma (Al-Rugeebah et al., 2019; Ghafouri-Fard and Taheri, 2019). Moreover, MEG3 expression increases the sensitivity of cancer cells to different therapies. In ovarian cancer tumors, there is a correlation between the downregulation of MEG3 and the response to cisplatin chemotherapy (El-Khazragy et al., 2020). Furthermore, in small-cell lung cancer (SCLC) cells, MEG3 expression increases sensitivity to cisplatin (Sun et al., 2022). In breast cancer patients, a decrease in MEG3 expression was associated with methylation of the gene promoter of MEG3 (Li et al., 2020). Accordingly, in breast cancer cell lines, MEG3 expression increases sensitivity to paclitaxel (Figure 4) (Zhu et al., 2020).

## Conclusion

The number of lncRNAs that have been described as associated with genome stability has increased recently; however, the functional association is still unknown, and the role of lncRNAs inducing genomic instability has been poorly explored in several cancers. On the other hand, Its role in other hallmarks of cancer, such as uncontrolled proliferation, resisting cell death, and activating invasion and metastasis, is better understood. In addition, the lncRNAs have pleiotropic effects, which makes it difficult to determine if their activity in the mechanisms of resistance and sensitivity to cancer treatment is related to their function in the stability of the genome or is due to independent mechanisms influencing the development of cancer. A better understanding of lncRNAs functions in the process of carcinogenesis may provide new insights into cancer treatment and allow us to propose the lncRNAs as possible biomarkers in specific neoplasms for clinical prognosis.

## References

[B1] Aguilera A. Garcia-Muse T. (2012). R loops: From transcription byproducts to threats to genome stability. Mol. Cell 46 (2), 115–124. 10.1016/j.molcel.2012.04.009 22541554

[B2] Al-Rugeebah A. Alanazi M. Parine N. R. (2019). MEG3: An oncogenic long non-coding RNA in different cancers. Pathol. Oncol. Res. 25 (3), 859–874. 10.1007/s12253-019-00614-3 30793226

[B3] Altemose N. Logsdon G. A. Bzikadze A. V. Sidhwani P. Langley S. A. Caldas G. V. (2022). Complete genomic and epigenetic maps of human centromeres. Science 376 (6588), eabl4178. 10.1126/science.abl4178 35357911 PMC9233505

[B4] Arnoult N. Van Beneden A. Decottignies A. (2012). Telomere length regulates TERRA levels through increased trimethylation of telomeric H3K9 and HP1α. Nat. Struct. Mol. Biol. 19 (9), 948–956. 10.1038/nsmb.2364 22922742

[B5] Arora R. Azzalin C. M. (2015). Telomere elongation chooses TERRA ALTernatives. RNA Biol. 12 (9), 938–941. 10.1080/15476286.2015.1065374 26158306 PMC4615670

[B6] Arora R. Lee Y. Wischnewski H. Brun C. M. Schwarz T. Azzalin C. M. (2014). RNaseH1 regulates TERRA-telomeric DNA hybrids and telomere maintenance in ALT tumour cells. Nat. Commun. 5, 5220. 10.1038/ncomms6220 25330849 PMC4218956

[B7] Azzalin C. M. Lingner J. (2008). Telomeres: The silence is broken. Cell Cycle 7 (9), 1161–1165. 10.4161/cc.7.9.5836 18418035

[B8] Azzalin C. M. Reichenbach P. Khoriauli L. Giulotto E. Lingner J. (2007). Telomeric repeat containing RNA and RNA surveillance factors at mammalian chromosome ends. Science 318 (5851), 798–801. 10.1126/science.1147182 17916692

[B9] Bakhoum S. F. Ngo B. Laughney A. M. Cavallo J. A. Murphy C. J. Ly P. (2018). Chromosomal instability drives metastasis through a cytosolic DNA response. Nature 553 (7689), 467–472. 10.1038/nature25432 29342134 PMC5785464

[B10] Balk B. Dees M. Bender K. Luke B. (2014). The differential processing of telomeres in response to increased telomeric transcription and RNA-DNA hybrid accumulation. RNA Biol. 11 (2), 95–100. 10.4161/rna.27798 24525824 PMC3973735

[B11] Blasco M. A. (2005). Telomeres and human disease: Ageing, cancer and beyond. Nat. Rev. Genet. 6 (8), 611–622. 10.1038/nrg1656 16136653

[B12] Bouzinba-Segard H. Guais A. Francastel C. (2006). Accumulation of small murine minor satellite transcripts leads to impaired centromeric architecture and function. Proc. Natl. Acad. Sci. U. S. A. 103 (23), 8709–8714. 10.1073/pnas.0508006103 16731634 PMC1482643

[B13] Bury L. Moodie B. Ly J. McKay L. S. Miga K. H. Cheeseman I. M. (2020). Alpha-satellite RNA transcripts are repressed by centromere-nucleolus associations Elife, 9, e59770. 10.7554/eLife.59770 33174837 PMC7679138

[B14] Caceres-Gutierrez R. E. Andonegui M. A. Oliva-Rico D. A. Gonzalez-Barrios R. Luna F. Arriaga-Canon C. (2022). Proteasome inhibition alters mitotic progression through the upregulation of centromeric alpha-Satellite RNAs. FEBS J. 289 (7), 1858–1875. 10.1111/febs.16261 34739170 PMC9299679

[B15] Chen B. Dragomir M. P. Fabris L. Bayraktar R. Knutsen E. Liu X. (2020). The long noncoding RNA CCAT2 induces chromosomal instability through BOP1-AURKB signaling. Gastroenterology 159 (6), 2146–2162. 10.1053/j.gastro.2020.08.018 32805281 PMC7725986

[B16] Chen L. Yuan D. Yang Y. Ren M. (2019). LincRNA-p21 enhances the sensitivity of radiotherapy for gastric cancer by targeting the beta-catenin signaling pathway. J. Cell. Biochem. 120 (4), 6178–6187. 10.1002/jcb.27905 30484893

[B17] Chuang T. C. Moshir S. Garini Y. Chuang A. Y. C. Young I. T. Vermolen B. (2004). The three-dimensional organization of telomeres in the nucleus of mammalian cells. BMC Biol. 2, 12. 10.1186/1741-7007-2-12 15176976 PMC425602

[B18] Deng Z. Wang Z. Xiang C. Molczan A. Baubet V. Conejo-Garcia J. (2012). Formation of telomeric repeat-containing RNA (TERRA) foci in highly proliferating mouse cerebellar neuronal progenitors and medulloblastoma. J. Cell Sci. 125, 4383–4394. 10.1242/jcs.108118 22641694 PMC3516443

[B19] Dimitrova N. Zamudio J. R. Jong R. M. Soukup D. Resnick R. Sarma K. (2014). LincRNA-p21 activates p21 in cis to promote Polycomb target gene expression and to enforce the G1/S checkpoint. Mol. Cell 54 (5), 777–790. 10.1016/j.molcel.2014.04.025 24857549 PMC4103188

[B20] El-Khazragy N. Mohammed H. F. Yassin M. Elghoneimy K. K. Bayoumy W. Hewety A. (2020). Tissue-based long non-coding RNAs "PVT1, TUG1 and MEG3" signature predicts Cisplatin resistance in ovarian Cancer. Genomics 112 (6), 4640–4646. 10.1016/j.ygeno.2020.08.005 32781203

[B21] Elguindy M. M. Kopp F. Goodarzi M. Rehfeld F. Thomas A. Chang T. C. (2019). PUMILIO, but not RBMX, binding is required for regulation of genomic stability by non-coding RNA NORAD. Elife, 8, e48625. 10.7554/eLife.48625 31343408 PMC6677556

[B22] Enukashvily N. I. Semenova N. Chubar A. V. Ostromyshenskii D. I. Gushcha E. A. Gritsaev S. (2022). Pericentromeric non-coding DNA transcription is associated with niche impairment in patients with ineffective or partially effective multiple myeloma treatment. Int. J. Mol. Sci. 23 (6), 3359. 10.3390/ijms23063359 35328779 PMC8951104

[B23] Esfandi F. Taheri M. Kholghi Oskooei V. Ghafouri-Fard S. (2019). Long non-coding RNAs expression in gastric cancer. J. Cell. Biochem. 120 (8), 13802–13809. 10.1002/jcb.28653 30938855

[B24] Eymery A. Horard B. El Atifi-Borel M. Fourel G. Berger F. Vitte A. L. (2009). A transcriptomic analysis of human centromeric and pericentric sequences in normal and tumor cells. Nucleic Acids Res. 37 (19), 6340–6354. 10.1093/nar/gkp639 19720732 PMC2770647

[B25] Farnung B. O. Brun C. M. Arora R. Lorenzi L. E. Azzalin C. M. (2012). Telomerase efficiently elongates highly transcribing telomeres in human cancer cells. PLoS One 7 (4), e35714. 10.1371/journal.pone.0035714 22558207 PMC3338753

[B26] Feng X. Yang S. Zhou S. Deng S. Xie Y. (2020). Long non-coding RNA DDX11-AS1 promotes non-small cell lung cancer development via regulating PI3K/AKT signalling. Clin. Exp. Pharmacol. Physiol. 47 (9), 1622–1631. 10.1111/1440-1681.13325 32298476

[B27] Feng Y. Wu M. Hu S. Peng X. Chen F. (2020). LncRNA DDX11-AS1: A novel oncogene in human cancer. Hum. Cell 33 (4), 946–953. 10.1007/s13577-020-00409-8 32772230

[B28] Flynn R. L. Centore R. C. O'Sullivan R. J. Rai R. Tse A. Songyang Z. (2011). TERRA and hnRNPA1 orchestrate an RPA-to-POT1 switch on telomeric single-stranded DNA. Nature 471 (7339), 532–536. 10.1038/nature09772 21399625 PMC3078637

[B29] Flynn R. L. Cox K. E. Jeitany M. Wakimoto H. Bryll A. R. Ganem N. J. (2015). Alternative lengthening of telomeres renders cancer cells hypersensitive to ATR inhibitors. Science 347 (6219), 273–277. 10.1126/science.1257216 25593184 PMC4358324

[B30] Fukagawa T. Earnshaw W. C. (2014). The centromere: Chromatin foundation for the kinetochore machinery. Dev. Cell 30 (5), 496–508. 10.1016/j.devcel.2014.08.016 25203206 PMC4160344

[B31] Gala K. Khattar E. (2021). Long non-coding RNAs at work on telomeres: Functions and implications in cancer therapy. Cancer Lett. 502, 120–132. 10.1016/j.canlet.2020.12.036 33450357

[B32] Ghafouri-Fard S. Azimi T. Hussen B. M. Abak A. Taheri M. Dilmaghani N. A. (2021). Non-coding RNA activated by DNA damage: Review of its roles in the carcinogenesis. Front. Cell Dev. Biol. 9, 714787. 10.3389/fcell.2021.714787 34485302 PMC8415109

[B33] Ghafouri-Fard S. Sohrabi B. Hussen B. M. Mehravaran E. Jamali E. Arsang-Jang S. (2022). Down-regulation of MEG3, PANDA and CASC2 as p53-related lncRNAs in breast cancer. Breast Dis. 41 (1), 137–143. 10.3233/BD-210069 35034894

[B34] Ghafouri-Fard S. Taheri M. (2019). Maternally expressed gene 3 (MEG3): A tumor suppressor long non coding RNA. Biomed. Pharmacother. 118, 109129. 10.1016/j.biopha.2019.109129 31326791

[B35] Guo J. Xiao D. Lin Z. Sui C. (2022). The long non-coding RNA PANDAR regulates cell proliferation and epithelial-to-mesenchymal transition in glioma. Histol. Histopathol., 18511. 10.14670/HH-18-511 36073766

[B36] Han T. Wu Y. Hu X. Chen Y. Jia W. He Q. (2020). NORAD orchestrates endometrial cancer progression by sequestering FUBP1 nuclear localization to promote cell apoptosis. Cell Death Dis. 11 (6), 473. 10.1038/s41419-020-2674-y 32555178 PMC7303217

[B37] Hanahan D. Weinberg R. A. (2011). Hallmarks of cancer: The next generation. Cell 144 (5), 646–674. 10.1016/j.cell.2011.02.013 21376230

[B38] He Y. H. Yeh M. H. Chen H. F. Wang T. S. Wong R. H. Wei Y. L. (2021). ERα determines the chemo-resistant function of mutant p53 involving the switch between lincRNA-p21 and DDB2 expressions. Mol. Ther. Nucleic Acids 25, 536–553. 10.1016/j.omtn.2021.07.022 34589276 PMC8463322

[B39] Hedouin S. Grillo G. Ivkovic I. Velasco G. Francastel C. (2017). CENP-A chromatin disassembly in stressed and senescent murine cells. Sci. Rep. 7, 42520. 10.1038/srep42520 28186195 PMC5301216

[B40] Heidenreich B. Kumar R. (2017). TERT promoter mutations in telomere biology. Mutat. Res. 771, 15–31. 10.1016/j.mrrev.2016.11.002 28342451

[B41] Huang H. W. Xie H. Ma X. ZhaoF., Gao Y. (2017). Upregulation of LncRNA PANDAR predicts poor prognosis and promotes cell proliferation in cervical cancer. Eur. Rev. Med. Pharmacol. Sci. 21 (20), 4529–4535. 29131264

[B42] Huang Y. Yi Q. Feng J. Xie W. Sun W. Sun W. (2022). The role of lincRNA-p21 in regulating the biology of cancer cells. Hum. Cell 35 (6), 1640–1649. 10.1007/s13577-022-00768-4 35969349

[B43] Huarte M. Guttman M. Feldser D. Garber M. Koziol M. J. Kenzelmann-Broz D. (2010). A large intergenic non-coding RNA induced by p53 mediates global gene repression in the p53 response. Cell 142 (3), 409–419. 10.1016/j.cell.2010.06.040 20673990 PMC2956184

[B44] Hung T. Wang Y. Lin M. F. Koegel A. K. Kotake Y. Grant G. D. (2011). Extensive and coordinated transcription of non-coding RNAs within cell-cycle promoters. Nat. Genet. 43 (7), 621–629. 10.1038/ng.848 21642992 PMC3652667

[B45] Islam F. Zhou Y. Lam A. K. (2022). Long non-coding RNAs profiling using microarray in papillary thyroid carcinoma. Methods Mol. Biol. 2534, 135–148. 10.1007/978-1-0716-2505-7_10 35670973

[B46] Kanne J. Hussong M. Isensee J. Munoz-Lopez A. Wolffgramm J. HeB F. (2021). Pericentromeric Satellite III transcripts induce etoposide resistance. Cell Death Dis. 12 (6), 530. 10.1038/s41419-021-03810-9 34031359 PMC8144429

[B47] Lee S. Kopp F. Chang T. C. Sataluri A. Chen B. Sivakumar S. (2016). Non-coding RNA NORAD regulates genomic stability by sequestering PUMILIO proteins. Cell 164 (1-2), 69–80. 10.1016/j.cell.2015.12.017 26724866 PMC4715682

[B48] Li H. Wang P. Liu J. Liu W. Wu X. Ding J. (2020). Hypermethylation of lncRNA MEG3 impairs chemosensitivity of breast cancer cells. J. Clin. Lab. Anal. 34 (9), e23369. 10.1002/jcla.23369 32618397 PMC7521317

[B49] Liu Q. Xiao Y. Cai P. Li J. Li D. (2019). Long non-coding RNA DINO (damage induced non-coding) represses the development of gastric cancer by modulating p21 and Bcl-2 Associated X Protein (Bax) expression. J. Cell. Biochem. 120, 11190–11195. 10.1002/jcb.28394 30775800

[B50] Longhese M. P. Bonetti D. Manfrini N. Clerici M. (2010). Mechanisms and regulation of DNA end resection. EMBO J. 29 (17), 2864–2874. 10.1038/emboj.2010.165 20647996 PMC2944052

[B51] Maicher A. Lockhart A. Luke B. (2014). Breaking new ground: Digging into TERRA function. Biochim. Biophys. Acta 1839 (5), 387–394. 10.1016/j.bbagrm.2014.03.012 24698720

[B52] Marchese F. P. Grossi E. Marin-Bejar O. Bharti S. K. Raimondi I. Gonzalez J. (2016). A long non-coding RNA regulates sister chromatid cohesion. Mol. Cell 63 (3), 397–407. 10.1016/j.molcel.2016.06.031 27477908 PMC5893147

[B53] Marney C. B. Anderson E. S. Adnan M. Peng K. L. Hu Y. Weinhold N. (2021). p53-intact cancers escape tumor suppression through loss of long non-coding RNA Dino. Cell Rep. 35 (13), 109329. 10.1016/j.celrep.2021.109329 34192538 PMC8287872

[B54] Marney C. B. Anderson E. S. Baum R. Schmitt A. M. (2022). A unique spectrum of spontaneous tumors in dino knockout mice identifies tissue-specific requirements for tumor suppression. Cells 11 (11), 1818. 10.3390/cells11111818 35681513 PMC9180304

[B55] Mei Y. Deng Z. Vladimirova O. Gulve N. Johnson F. B. Drosopoulos W. C. (2021). TERRA G-quadruplex RNA interaction with TRF2 GAR domain is required for telomere integrity. Sci. Rep. 11 (1), 3509. 10.1038/s41598-021-82406-x 33568696 PMC7876106

[B56] Munschauer M. Nguyen C. T. Sirokman K. Hartigan C. R. Hogstrom L. Engreitz J. M. (2018). The NORAD lncRNA assembles a topoisomerase complex critical for genome stability. Nature 561 (7721), 132–136. 10.1038/s41586-018-0453-z 30150775

[B57] Nergadze S. G. Farnung B. O. Wischnewski H. Khoriauli L. Vitelli V. Chawla R. (2009). CpG-island promoters drive transcription of human telomeres. RNA 15 (12), 2186–2194. 10.1261/rna.1748309 19850908 PMC2779677

[B58] Oh B. K. Um T. H. Choi G. H. Park Y. N. (2011). Frequent changes in subtelomeric DNA methylation patterns and its relevance to telomere regulation during human hepatocarcinogenesis. Int. J. Cancer 128 (4), 857–868. 10.1002/ijc.25398 20473888

[B59] Oliva-Rico D. Fabian-Morales E. Caceres-Gutierrez R. E. Gudino A. Cisneros-Soberanis F. Dominguez J. (2022). Methylation of subtelomeric chromatin modifies the expression of the lncRNA TERRA, disturbing telomere homeostasis. Int. J. Mol. Sci. 23 (6), 3271. 10.3390/ijms23063271 35328692 PMC8955364

[B60] Oliva-Rico D. Herrera L. A. (2017). Regulated expression of the lncRNA TERRA and its impact on telomere biology. Mech. Ageing Dev. 167, 16–23. 10.1016/j.mad.2017.09.001 28888705

[B61] Peng C. Hu W. Weng X. Tong R. Cheng S. Ding C. (2017). Over expression of long non-coding RNA PANDA promotes hepatocellular carcinoma by inhibiting senescence associated inflammatory factor IL8. Sci. Rep. 7 (1), 4186. 10.1038/s41598-017-04045-5 28646235 PMC5482898

[B62] Peng W. Fan H. (2015). Long non-coding RNA PANDAR correlates with poor prognosis and promotes tumorigenesis in hepatocellular carcinoma. Biomed. Pharmacother. 72, 113–118. 10.1016/j.biopha.2015.04.014 26054684

[B63] Pirlog R. Drula R. Nutu A. Calin G. A. Berindan-Neagoe I. (2021). The roles of the colon cancer associated transcript 2 (CCAT2) long non-coding RNA in cancer: A comprehensive characterization of the tumorigenic and molecular functions. Int. J. Mol. Sci. 22 (22), 12491. 10.3390/ijms222212491 34830370 PMC8620102

[B64] Puvvula P. K. Desetty R. D. Pineau P. Marchio A. Moon A. Dejean A. (2014). Long non-coding RNA PANDA and scaffold-attachment-factor SAFA control senescence entry and exit. Nat. Commun. 5, 5323. 10.1038/ncomms6323 25406515 PMC4263151

[B65] Qing Y. Li Q. Zhao L. Y. Shi P. Shan J. L. Zhang W. (2021). LncRNA-PANDAR regulates the progression of thyroid carcinoma by targeting miR-637/KLK4. J. Cancer 12 (19), 5879–5887. 10.7150/jca.55181 34476001 PMC8408101

[B66] Redis R. S. Sieuwerts A. M. Look M. P. Tudoran O. Ivan C. Spizzo R. (2013). CCAT2, a novel long non-coding RNA in breast cancer: Expression study and clinical correlations. Oncotarget 4 (10), 1748–1762. 10.18632/oncotarget.1292 24077681 PMC3858561

[B67] Rivandi M. Pasdar A. Hamzezadeh L. Tajbakhsh A. Seifi S. Moetamani-Ahmadi M. (2019). The prognostic and therapeutic values of long non-coding RNA PANDAR in colorectal cancer. J. Cell. Physiol. 234 (2), 1230–1236. 10.1002/jcp.27136 30191971

[B68] SamudyataCastelo-Branco G. Bonetti A. (2018). Birth, coming of age and death: The intriguing life of long non-coding RNAs. Semin. Cell Dev. Biol. 79, 143–152. 10.1016/j.semcdb.2017.11.012 29133230

[B69] Sansregret L. Vanhaesebroeck B. Swanton C. (2018). Determinants and clinical implications of chromosomal instability in cancer. Nat. Rev. Clin. Oncol. 15 (3), 139–150. 10.1038/nrclinonc.2017.198 29297505

[B70] Santibanez-Andrade M. Sanchez-Perez Y. Chirino Y. I. Morales-Barcenas R. Garcia-Cuellar C. M. (2021). Long non-coding RNA NORAD upregulation induced by airborne particulate matter (PM10) exposure leads to aneuploidy in A549 lung cells. Chemosphere 266, 128994. 10.1016/j.chemosphere.2020.128994 33250223

[B71] Schmitt A. M. Garcia J. T. Hung T. Flynn R. A. Shen Y. Qu K. (2016). An inducible long non-coding RNA amplifies DNA damage signaling. Nat. Genet. 48 (11), 1370–1376. 10.1038/ng.3673 27668660 PMC5083181

[B72] Shen Y. Liu Y. Sun T. Yang W. (2017). LincRNA-p21 knockdown enhances radiosensitivity of hypoxic tumor cells by reducing autophagy through HIF-1/Akt/mTOR/P70S6K pathway. Exp. Cell Res. 358 (2), 188–198. 10.1016/j.yexcr.2017.06.016 28689810

[B73] Shi M. Zhang X. Y. Yu H. Xiang S. H. Xu L. Wei J. (2017). DDX11-AS1 as potential therapy targets for human hepatocellular carcinoma. Oncotarget 8 (27), 44195–44202. 10.18632/oncotarget.17409 28496001 PMC5546473

[B74] Shi W. Wang Q. Bian Y. Fan Y. Zhou Y. Feng T. (2019). Long non-coding RNA PANDA promotes esophageal squamous carcinoma cell progress by dissociating from NF-YA but interact with SAFA. Pathol. Res. Pract. 215 (10), 152604. 10.1016/j.prp.2019.152604 31495606

[B75] Smirnova A. Gamba R. Khoriauli L. Vitelli V. Nergadze S. G. Giulotto E. (2013). TERRA expression levels do not correlate with telomere length and radiation sensitivity in human cancer cell lines. Front. Oncol. 3, 115. 10.3389/fonc.2013.00115 23717814 PMC3650684

[B76] Soghli N. Yousefi T. Abolghasemi M. Qujeq D. (2021). NORAD, a critical long non-coding RNA in human cancers. Life Sci. 264, 118665. 10.1016/j.lfs.2020.118665 33127516

[B77] Song W. Qian Y. Zhang M. H. Wang H. Wen X. Yang X. Z. (2020). The long non-coding RNA DDX11-AS1 facilitates cell progression and oxaliplatin resistance via regulating miR-326/IRS1 axis in gastric cancer. Eur. Rev. Med. Pharmacol. Sci. 24 (6), 3049–3061. 10.26355/eurrev_202003_20669 32271422

[B78] Sun Y. Hao G. Zhuang M. Lv H. Liu C. Su K. (2022). MEG3 LncRNA from exosomes released from cancer-associated fibroblasts enhances cisplatin chemoresistance in SCLC via a MiR-15a-5p/CCNE1 Axis. Yonsei Med. J. 63 (3), 229–240. 10.3349/ymj.2022.63.3.229 35184425 PMC8860932

[B79] Tan B. S. Yang M. C. Singh S. Chou Y. C. Chen H. Y. Wang M. Y. (2019). LncRNA NORAD is repressed by the YAP pathway and suppresses lung and breast cancer metastasis by sequestering S100P. Oncogene 38 (28), 5612–5626. 10.1038/s41388-019-0812-8 30967631

[B80] Tanaka K. Hirota T. (2016). Chromosomal instability: A common feature and a therapeutic target of cancer. Biochim. Biophys. Acta 1866 (1), 64–75. 10.1016/j.bbcan.2016.06.002 27345585

[B81] Taniue K. Akimitsu N. (2021). The functions and unique features of LncRNAs in cancer development and tumorigenesis. Int. J. Mol. Sci. 22 (2), E632. 10.3390/ijms22020632 PMC7826647 33435206

[B82] Tian J. B. Cao L. Dong G. L. (2019). Long non-coding RNA DDX11-AS1 induced by YY1 accelerates colorectal cancer progression through targeting miR-873/CLDN7 axis. Eur. Rev. Med. Pharmacol. Sci. 23 (13), 5714–5729. 10.26355/eurrev_201907_18309 31298324

[B83] Ting D. T. Lipson D. Paul S. Brannigan B. W. Akhavanfard S. Coffman E. J. (2011). Aberrant overexpression of satellite repeats in pancreatic and other epithelial cancers. Science 331 (6017), 593–596. 10.1126/science.1200801 21233348 PMC3701432

[B84] Toubiana S. Selig S. (2018). DNA:RNA hybrids at telomeres - when it is better to be out of the (R) loop. FEBS J. 285 (14), 2552–2566. 10.1111/febs.14464 29637701

[B85] Turajlic S. Sottoriva A. Graham T. Swanton C. (2019). Resolving genetic heterogeneity in cancer. Nat. Rev. Genet. 20 (7), 404–416. 10.1038/s41576-019-0114-6 30918367

[B86] Valgardsdottir R. Chiodi I. Giordano M. Rossi A. Bazzini S. Ghigna C. (2008). Transcription of Satellite III non-coding RNAs is a general stress response in human cells. Nucleic Acids Res. 36 (2), 423–434. 10.1093/nar/gkm1056 18039709 PMC2241877

[B87] Vargas-Rondon N. Villegas V. E. Rondon-Lagos M. (2017). The role of chromosomal instability in cancer and therapeutic responses. Cancers (Basel) 10 (1), E4. 10.3390/cancers10010004 PMC5789354 29283387

[B88] Wang C. Zhao L. Lu S. (2015). Role of TERRA in the regulation of telomere length. Int. J. Biol. Sci. 11 (3), 316–323. 10.7150/ijbs.10528 25678850 PMC4323371

[B89] Wang X. Ruan Y. Wang X. Zhao W. Jiang Q. Jiang C. (2017). Long intragenic non-coding RNA lincRNA-p21 suppresses development of human prostate cancer. Cell Prolif. 50 (2), e12318. 10.1111/cpr.12318 27976428 PMC6529152

[B90] Wang X. Xu Y. Wang X. Jiang C. Han S. Dong K. (2017). LincRNA-p21 suppresses development of human prostate cancer through inhibition of PKM2. Cell Prolif. 50 (6), e12395. 10.1111/cpr.12395 28994148 PMC6529145

[B91] Wang Y. Zhang M. Xu H. Wang Y. Li Z. Chang Y. (2017). Discovery and validation of the tumor-suppressive function of long non-coding RNA PANDA in human diffuse large B-cell lymphoma through the inactivation of MAPK/ERK signaling pathway. Oncotarget 8 (42), 72182–72196. 10.18632/oncotarget.20053 29069778 PMC5641121

[B92] Xiang Z. Lv Q. Zhang Y. Chen X. Guo R. Liu S. (2022). Long non-coding RNA DDX11-AS1 promotes the proliferation and migration of glioma cells by combining with HNRNPC. Mol. Ther. Nucleic Acids 28, 601–612. 10.1016/j.omtn.2022.04.016 35614994 PMC9109126

[B93] Yehezkel S. Segev Y. Viegas-Pequignot E. Skorecki K. Selig S. (2008). Hypomethylation of subtelomeric regions in ICF syndrome is associated with abnormally short telomeres and enhanced transcription from telomeric regions. Hum. Mol. Genet. 17 (18), 2776–2789. 10.1093/hmg/ddn177 18558631

[B94] Yu Y. Chen F. Jin Y. Yang Y. Wang S. Zhang J. (2020). Downregulated NORAD in neuroblastoma promotes cell proliferation via chromosomal instability and predicts poor prognosis. Acta Biochim. Pol. 67 (4), 595–603. 10.18388/abp.2020_5454 33326736

[B95] Zhang H. Lin J. Chen J. Gu W. Mao Y. Wang H. (2020). DDX11-AS1 contributes to osteosarcoma progression via stabilizing DDX11. Life Sci. 254, 117392. 10.1016/j.lfs.2020.117392 32014424

[B96] Zhang L. Wang Y. Xia S. Yang L. Wu D. Zhou Y. (2020). Long non-coding RNA PANDAR inhibits the development of lung cancer by regulating autophagy and apoptosis pathways. J. Cancer 11 (16), 4783–4790. 10.7150/jca.45291 32626525 PMC7330687

[B97] Zhang S. Jiang H. Xu Z. Jiang Y. She Y. Huang X. (2019). The resistance of esophageal cancer cells to paclitaxel can be reduced by the knockdown of long non-coding RNA DDX11-AS1 through TAF1/TOP2A inhibition. Am. J. Cancer Res. 9 (10), 2233–2248. 31720085 PMC6834486

[B98] Zhu M. Wang F. Mi H. Li L. Wang J. Han M. (2020). Long non-coding RNA MEG3 suppresses cell proliferation, migration and invasion, induces apoptosis and paclitaxel-resistance via miR-4513/PBLD axis in breast cancer cells. Cell Cycle 19 (23), 3277–3288. 10.1080/15384101.2020.1839700 33121324 PMC7751664

[B99] Zhu Q. Pao G. M. Huynh A. M. Suh H. Tonnu N. Nederlof P. M. (2011). BRCA1 tumour suppression occurs via heterochromatin-mediated silencing. Nature 477 (7363), 179–184. 10.1038/nature10371 21901007 PMC3240576

[B100] Zou Y. Zhong Y. Wu J. Xiao H. Zhang X. Liao X. (2018). Long non-coding pandar as a novel biomarker in human cancer: A systematic review. Cell Prolif. 51 (1). 10.1111/cpr.12422 PMC6528858 29226461

